# Memory-Guided Saccades in Subacute and Chronic Stroke: Secondary Data Analysis of the N-PEP-12 Clinical Study

**DOI:** 10.3390/biomedicines12081678

**Published:** 2024-07-26

**Authors:** Emanuel Ștefănescu, Maria Balea, Vlad-Florin Chelaru, Nicoleta Jemna, Olivia Verișezan Roșu, Anamaria Truță, Adina Dora Stan, Diana Chira, Ștefan Strilciuc, Dafin Mureșanu

**Affiliations:** 1 Department of Neuroscience, Iuliu Hațieganu University of Medicine and Pharmacy, 400012 Cluj-Napoca, Romania; emanuel.stefanescu@brainscience.ro (E.Ș.); dafinm@ssnn.ro (D.M.); 2 RoNeuro Institute for Neurological Research and Diagnostic, 400364 Cluj-Napoca, Romania; 3 Research Center for Functional Genomics, Biomedicine, and Translational Medicine, Iuliu Hațieganu University of Medicine and Pharmacy, 400012 Cluj-Napoca, Romania

**Keywords:** ischemic stroke, neuropsychological assessment, eye tracking

## Abstract

Background: Ischemic stroke (IS) often leads to cognitive and motor impairments. This study aimed to investigate whether Memory-Guided Saccade Tasks (MGSTs) could be used to assess cognitive function in stroke patients. Methods: A secondary data analysis was conducted on 62 individuals with supratentorial IS. Eye-tracking metrics from MGST were correlated with established neuropsychological assessments, including the Montreal Cognitive Assessment (MoCA) and Hospital Anxiety and Depression Scale (HADS). Results: Age correlated negatively with memory-guided saccade (MGS) accuracy (ρ = −0.274) and positively with late errors (ρ = 0.327). Higher Montreal Cognitive Assessment (MoCA) scores were associated with faster corrective saccades (ρ = 0.259). Increased anxiety (HADS-A) and depression (HADS-D) levels correlated with higher early error rates (ρ = 0.325 and ρ = 0.311, respectively). The Color Trails Test and Digit Span test performance also correlated with various MGS parameters. Conclusions: While some correlations were found between cognitive measures and eye-tracking metrics, further research is needed to validate MGST as a tool for cognitive assessment in a more homogenous stroke population.

## 1. Introduction

Stroke is a widespread health burden worldwide, recognized as a leading cause of disability and mortality and associated with a wide range of cognitive and motor impairments, with a significant impact on patient’s functional outcomes, abilities, and quality of life [1,2,3]. Ischemic stroke (IS), particularly when it occurs in the supratentorial region of the brain, represents a major medical challenge, due to its significant long-term complications and deficits [4,5,6]. The clinical assessment of post-stroke patients is mainly focused on physical and cognitive impairments, while deficits in eye movement control, specifically saccadic eye movements, are prevalent but frequently underexplored [7,8,9].

Assessing the cognitive and psychological profile of patients diagnosed with IS is considered essential in designing effective, personalized therapeutic and targeted rehabilitation strategies. The clinical use of Memory-Guided Saccadic Tasks (MGST), Montreal Cognitive Assessment (MoCA), the Hospital Anxiety and Depression Scale (HADS), Wechsler Adult Intelligence Scale (WAIS-III) subtests, and the Color Trails Test provides a comprehensive evaluation of these domains in supratentorial IS patients and offers detailed insights into cognitive deficits, emotional states, and functional impairments in patients diagnosed with IS [2,10,11,12,13,14,15].

The Memory-Guided Saccadic Task (MGST) is a neuropsychological assessment tool used to evaluate the cortical functions related to ocular motor control and spatial working memory. MGST might be considered an essential investigation tool in the assessment of cognitive and neurological functions, due to its possible use in exploring the relationship between visual target retention and saccadic eye movements as a characteristic of visual perception and attention (Table 1). Integrating the MGST into clinical practice increases the accuracy of IS assessment and might be useful in defining targeted therapeutic strategies and personalized rehabilitation strategies, resulting in improved patient care and better clinical outcomes [15,16,17,18,19,20,21].

Cognition results from the dynamic interplay between mechanisms such as excitation–inhibition and synchronization–desynchronization, driven by the organization and interaction of multiple brain systems and subsystems [22]. The N-PEP study evaluated the effects of N-Pep-12 dietary supplementation on neurorecovery in middle-aged and older adults with cognitive impairment following an ischemic stroke. The study demonstrated the benefits of dietary supplementation with N-Pep-12 for enhancing neurorecovery after supratentorial ischemic stroke, as evidenced by conventional neuropsychological evaluations and qEEG indicators [23,24].

This secondary data analysis of the N-PEP-12 study aimed to determine whether eye-tracking metrics from a Memory-Guided Saccadic Task could effectively assess cognitive function in stroke patients, by examining their correlations with standard psychological evaluations.

**Table 1:** MGST assessment of cognitive and neurological functions post-IS [15,17,18,19,20,21,25].

Evaluated Domains	Clinical Assessments
Neurological Integrity	-Functional status of frontal and parietal lobes post stroke
Executive Function	-Evaluates planning and execution of eye movements
Working Memory	-Assesses patients’ ability to hold and manipulate spatial information

## 2. Materials and Methods

The current study is a secondary data analysis of a cohort of 62 individuals who experienced supratentorial ischemic stroke. Data were collected from participants in the clinical trial “Combined Neuropsychological, Neurophysiological, and Psychophysiological Assessment of the Effects of N-Pep-12 on Neurorecovery in Patients After Ischemic Stroke” that took place at the RoNeuro Institute for Neurological Research and Diagnostics in Cluj-Napoca, Romania (protocol available at https://doi.org/10.1186/ISRCTN10702895 accessed on 20 June 2024). The investigation adhered to the ethical standards outlined in the Declaration of Helsinki. Each participant provided signed informed consent prior to enrollment. The Ethics Committee of the “Iuliu Hațieganu” University of Medicine and Pharmacy Cluj-Napoca, Romania, reviewed and approved the research protocol (reference number: 507/10.12.2015; Ethics Committee of the Iuliu Hațieganu University of Medicine and Pharmacy, 8 Babeş Street, 400012 Cluj-Napoca, Romania; +40-264-597-256; contact@umfcluj.ro). Subsequent amendments were approved under references 82/24.03.2016 and 104/12.02.2018. The initial approval date was 10 December 2015.

The investigation included participants aged 18–80 who were 30–120 days post supratentorial ischemic stroke, confirmed radiologically (CT or MRI). Participants had no significant pre-stroke disability, as indicated by a pre-stroke Modified Rankin Scale score and a minimum score of 2 on the Goodglass and Kaplan Communication Scale. Additionally, participants had not suffered any other stroke within the three months preceding the index stroke. Exclusion criteria were pre-existing and active major neurological or psychiatric disease (depression, schizophrenia, bipolar disease, or dementia—IQCODE score > 3), advanced liver, kidney, cardiac, or pulmonary disease, terminal medical diagnosis consistent with survival of less than 1 year, females who were pregnant or lactating, major drug or alcohol dependency, injury of the writing hand, influencing cognitive or other outcome measures, neglect, hemianopsia, myopia > 3 dioptres, or glaucoma.

### 2.1. Study Procedures

The evaluation of all 62 stroke patients was conducted 30–120 days after stroke onset. Each subject was clinically examined by a trained neurologist and underwent a neuropsychological evaluation by a specialized psychologist.

Memory-guided saccades were evaluated during a task spanning approximately 5 min. For this purpose, the Tobii Tx300 screen-based dark pupil eye-tracking system (Tobii Technology, Stockholm, Sweden) was used. The visual stimulus sequence was generated using Tobii Studio 3.4.8. software and presented on a 23-inch display with a 16:9 aspect ratio, 1920 × 1080 pixel resolution, a refresh rate of 60 Hz, and 5 ms screen response time. The device provided an average gaze accuracy of 0.4° and a processing latency ranging from 1.0 to 3.3 ms.

Following a 9-point calibration stage performed by the eye tracking device, binocular recordings were made at a sample rate of 250 Hz. The experiment was conducted in a quiet and dark room to minimize external distractions. Participants were seated 65 cm from the screen in a comfortable, height-adjustable chair with a chin and forehead rest to limit head movement. The visual stimulus consisted of a red dot with a diameter that subtended a visual angle of 0.4° (luminance of 63 cd/m^2^), presented on a black background (luminance of 2.5 cd/m^2^)

**Figure 1:**
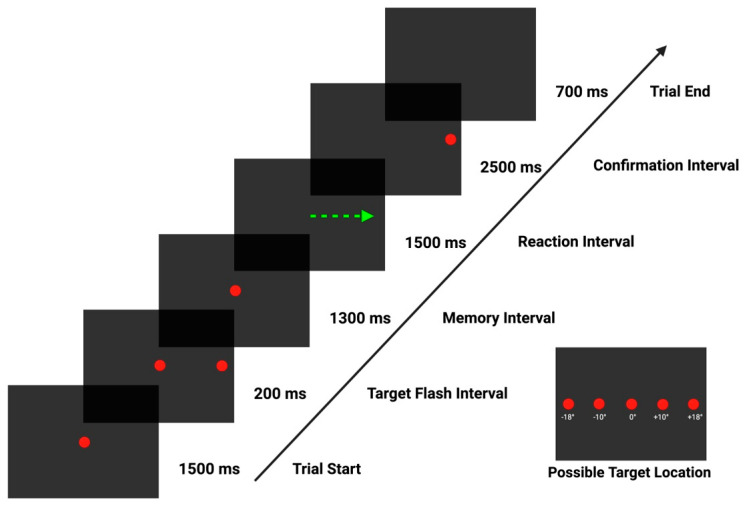
Memory-Guided Saccadic Task trial structure. The sequence starts with a central stimulus (red dot), displayed for 1500 ms. Next, a randomized left or right eccentric horizontal target flash is presented for 200 ms alongside the central dot (+/−10° or +/−18° from the center). This phase is referred to as the target flash interval. Then, the central stimulus is displayed for 1300 ms (memory interval). The central fixation dot disappears, leaving a black background for 1500 ms (reaction interval, green arrow). The trial continues with a 2500 ms confirmation interval, where a confirmation dot appears eccentrically, at the target flash location [26,27]. The trial ends with a 700 ms black background. The evaluation consisted of 40 trials, 10 for each target amplitude.

Each participant performed a Memory-Guided Saccadic Task evaluation (Figure 1). A briefing session was conducted with each participant before initiating the eye tracking evaluation, followed by a practice trial session to ensure a proper understanding of the evaluation. Participants were instructed to fixate on the central stimulus and to suppress any reflexive eye movement from the onset of the trial, during the target flash interval, and throughout the memory interval, for a total of 3000 ms. Subjects were requested to fixate on the remembered peripheral target position after central stimulus disappearance (i.e., “go” signal, marking the start of the reaction interval) until appearance of a dot (confirmation stimulus), thus eliciting a memory-guided saccade. At the start of the confirmation interval, subjects were instructed to voluntarily redirect their gaze from the remembered target location to the actual target location, correcting their gaze position by performing a visually guided saccade (VGS).

### 2.2. Data Processing

The built-in Tobii IV-T fixation filter was used for gap-fill interpolation sets at 75 ms. Eye selection was based on the average of both eyes, and noise reduction was disabled. The velocity window length was 20 ms, with the I-VT classifier threshold at 30°/s. Adjacent fixations were merged if the maximum time between them was 75 ms and the maximum angle between them was 0.5°. The minimum fixation duration was set at 60 ms [28,29]. Exported individual .tsv files for each recording were further processed using an in-house script with built-in R (version 4.3.2.).

To guarantee the perception of the onset of the target flash interval, we included only trials with no saccadic eye movements, no blinks, and whose subject gaze was located 100 pixels (~2.3°) left and right of the center of the screen 60 ms before the start of this interval. A minimum of 7 valid trials had to be completed by each subject for the data to be included in the analysis. In our analysis, we included only the first saccade made after target onset for both memory-guided saccades and visually guided saccades. Memory-guided saccades had a minimum amplitude of 1° and their starting point was within 100 pixels (~2.3°) to the left and right of the center of the screen. There were no saccades or blinks 100 ms prior to the analyzed saccade. Trials were divided into “near” and “far” categories based on the eccentricity of the lateral target location at two different amplitudes, 10° and 18° of visual angle from the center of the screen, respectively.

Latency, amplitude, duration, mean velocity, peak velocity, time to peak velocity and gain were determined for each valid saccadic eye movement. Latency is defined as the time interval, in milliseconds, between the disappearance of the central dot (“go” signal) and the start of the saccade. Amplitude is defined as the difference in degrees of visual angle between the position of the gaze at the start and end of the saccadic eye movement. Duration is defined as the time interval difference, in milliseconds, between the onset time and the offset time of the saccade. Mean velocity is defined as the average speed of the saccade, in °/ms. Peak velocity is the maximum velocity in °/ms of the saccadic eye movement. Gain was calculated from (amplitude saccade/amplitude target) × 100 [30,31]. We defined errors as saccades occurring after the appearance of the eccentric target, which were further classified as early errors (i.e., if they occurred in the first 300 ms) and late errors (i.e., for saccadic movements made after 300 ms). Mean and SD deviation across trials for each subject and each eccentricity (near and far) were computed for latency, amplitude, duration, mean velocity, peak velocity, time to peak velocity and gain. The total error rate was calculated as the sum of early and late errors [32]. Additional performance indicators are available in Table 2.

### 2.3. Neuropsychological Assessment

We used measurements from several neuropsychological evaluation scales available in the trial. MoCA, a widely used screening test for cognitive impairment, provided an overview of the subjects’ cognitive abilities [33]. HADS, Anxiety and Depression subscales, were used to assess anxiety and depression levels [34]. Color Trails Test (CTT), an alternative to the Trail Making Test (TMT), was used to assess attention and visual–spatial reasoning (both subtests were used, and the following outcomes were obtained: time in seconds to complete the test, number of errors, number of near misses, and number of prompts) [35]. The Digit Span Forward test was used to assess working memory, attention, and short-term memory, while the Digit Span Backward test focused on evaluating the subject’s capacity for sequencing and working memory [36]. For Processing Speed Index, Digit Symbol Coding and Symbol Search were used to assess the ability to accurately process visual information, as well as concentration, short term memory, and attention to detail [37].

Alongside the previously described eye-tracking parameters generated by the Memory-Guided Saccadic Task, we used the following psychological outcome variables: MoCA total score, total anxiety score (HADS-A), total depression score (HADS-D), Color Trails Test 1—time in seconds (CLRES01-1), errors (CLRES02-1), near misses (CLRES03-1), prompts (CLRES04-1), Color Trails Test 2—time in seconds (CLRES05-1), color errors (CLRES06-1), number errors (CLRES07-1), near misses (CLRES08-1), prompts (CLRES09-1), total score for each of the two Digit Span subtests (DGFRES-1 and DGBRES-1), Digit Symbol Coding—number correct (PSCNUM-1), Symbol Search—number correct (PSSCNUM-1), and number incorrect (PSSINUM-1).

For statistical analysis, we used R version 4.3.2. [38] with RStudio [39] along with the following libraries: “openxlsx” [40] to interact with spreadsheet files, and “ggplot” [41] and “patchwork” [42] to generate graphical representations. All analyses were conducted using a significance level of alpha = 0.05. Pearson and Spearman correlation analyses were performed to investigate the relationship between demographic variables, neuropsychological scores, and Memory-Guided Saccadic Task outcomes. To further assess the influence of age and other relevant variables on memory-guided saccades performance, we conducted a multiple regression analysis using the percentage of late errors (%LateErrorRate) as the dependent variable. The independent variables included age, gender, MOTS (Montreal Cognitive Assessment) score, HDTSA-1 (Hospital Anxiety and Depression Scale—Anxiety), HDTSD-1 (Hospital Anxiety and Depression Scale—Depression), CLRES01-1, and CLRES02-1. This analysis was performed using Python 3 and the following packages: statsmodels for regression analysis and diagnostic tests, matplotlib and seaborn for data visualization, and pandas for data manipulation. The assumptions of multiple regression (linearity, independence, homoscedasticity, normality of residuals, and multicollinearity) were checked and met. The Variance Inflation Factor (VIF) was calculated to assess multicollinearity [43,44,45,46].

Aggregated data used in the analyses are available at https://doi.org/10.7910/DVN/VQ2AEE, accessed on 20 June 2024. Individual mean values and standard deviations (SD) can be provided upon reasonable request.

## 3. Results

Although 121 individuals were initially enrolled in the primary study, only 62 were included in our analysis, based on the availability of recordings, non-compliance during recording sessions, device calibration difficulties, low-quality data with frequent signal loss during sessions, or a low count of correctly executed memory-guided saccadic eye movements. The demographic structure of our sample is available in Table 3.

### Correlation Analysis Results

Age demonstrated a significant positive moderate correlation with %LateErrorRate. A significant negative weak correlation was also observed between age and the %MGS. Additionally, age showed a positive weak correlation with the mean value of time to peak velocity of NEAR VGS. 

The MoCA total score demonstrated a significant positive weak correlation with the mean duration of NEAR VGS. Anxiety levels, measured by HADS–A, correlated positively yet moderately with the %EarlyErrorRate. HADS-A total score showed a weak correlation with the mean values of the mean and peak velocities of the NEAR MGS and with the mean value of the peak velocity for the MGS FAR. Lastly, anxiety total score showed a negative weak correlation with the standard deviation (SD) of the duration of the MGS FAR.

Depression levels, calculated with HADS-D, showed a positive moderate correlation with the %EarlyErrorRate and a positive weak correlation with %TotalErrorRate.

The time in seconds to complete the CTT-1 test showed a positive moderate correlation with the SD value for the mean velocity of the MGS NEAR and a positive weak correlation with the SD value for the peak velocity of the MGS NEAR. The number of errors in the CCT-1 test revealed negative moderate correlations with the MGS FAR SD of latency and mean velocity, as well as a negative weak correlation with the SD of peak velocity. Additionally, there were negative weak correlations with the VGS NEAR mean value of mean velocity and the SD of peak velocity, and negative moderate correlations with the SD of amplitude for both VGS NEAR and VGS FAR. Near misses during the CCT-1 test showed a positive weak correlation with MGS NEAR latency and a negative weak correlation with the mean value of peak velocity for MGS FAR targets. There were several positive correlations between the number of near misses in CTT-1 and VGS parameters: a moderate correlation with %CorrVGS and for VGS FAR, with mean values of amplitude, duration, mean velocity, and time to peak velocity. The number of prompts showed a positive weak correlation with the %EarlyErrorRate and a negative weak correlation with %MGS.

The time in seconds to complete the CTT-2 test correlated positively moderately with the standard deviation (SD) values of mean velocity of the MGS NEAR and negatively moderately with the SD values of mean velocity of MGS FAR. The number of color errors negatively correlated with both the mean and SD values of peak velocity of the MGS FAR. The number of prompts showed a positive moderate correlation with the %EarlyErrorRate.

The Digit Span Forward scores showed positive weak correlations with the SD values of MGS NEAR amplitude and gain.

The Digit Span Backward scores showed a negative moderate correlation with the mean value of time to peak velocity for the MGS NEAR and a positive moderate correlation with %CorrVGS.

Processing Speed Index—Symbol Search—Number Correct showed a negative weak correlation with MGS NEAR for mean values of time to peak velocity and SD values of mean velocity, and with MGS FAR for SD values of latency. Number Incorrect showed a negative weak correlation with MGS NEAR for SD values of duration and a positive moderate correlation with the mean value for time to peak velocity of the VGS NEAR. Table 4 presents all correlation results more clearly. Additional descriptive analysis of neuropsychological outcomes and saccade data is available in Supplementary Materials.

A multiple regression analysis was conducted to evaluate the effects of age, gender, and other relevant variables on %LateErrorRate. The overall regression model was statistically significant, F(7,54) = 1.909, *p* = 0.0859, and explained approximately 19.8% of the variance in %LateErrorRate (R2 = 0.198). The assumptions for multiple regression were tested and met.

The regression coefficient for age was positive and significant (B = 0.634, *p* = 0.018), indicating that for each additional year of age, the %LateErrorRate increased by approximately 0.63%. This suggests a clear age-related increase in the frequency of late errors during the memory-guided saccades task.

The coefficient for gender (B = 10.561, *p* = 0.087) was positive, indicating that males had a higher %LateErrorRate compared to females, although this result was borderline non-significant. This finding suggests a potential gender difference that warrants further investigation (Table 5).

## 4. Discussion

The primary objective of this secondary data analysis was to evaluate whether the outcomes of a Memory-Guided Saccadic Task assessment could serve as a potential candidate for cognitive function assessment in stroke patients. We looked at the correlations between conventional psychological evaluations and eye-tracking metrics derived from this task. 

Our findings indicate that age significantly impacts performance in Memory-Guided Saccadic Tasks. The percentage of late errors during the memorization phase increased noticeably with age. Additionally, older individuals showed a decline in peak velocity of the corrective visually guided saccades and a decrease in the overall percentage of valid memory-guided saccades.

The multiple regression analysis revealed that age had a significant positive effect on %LateErrorRate, suggesting that older individuals tend to have higher late error rates. This finding aligns with existing literature indicating that cognitive and motor functions may decline with age. Gender also showed a borderline significant effect, with males potentially having higher late error rates, although this result was not conclusive.

These findings are consistent with current research on eye movements in the elderly, across various contexts. Research has shown that saccade metrics, such as peak velocity, latency, and spatial accuracy, differ significantly between older adults with and without cognitive impairment, indicating the potential of saccades as a screening tool for mild cognitive impairment (MCI) and dementia [47]. The inhibitory control of reflexive saccadic eye movement might decreases with age when testing for anti-saccadic eye movements and memory-guided saccadic eye movements [48,49,50].

Enhanced cognitive ability, as indicated by higher MoCA scores, appears to be associated with higher peak velocities of the corrective saccades during the confirmation interval, from the fixated target location of the memory-guided saccade towards the actual NEAR target location. It has been demonstrated that an increase in mental workload decreases saccadic peak velocity [51]. This suggests that with better cognitive abilities, the subject might sustain higher mental workloads, as shown by higher peak velocities.

Higher anxiety levels of stroke patients were associated with an increased percentage of early errors, higher mean and peak velocities of memory-guided saccades for both eccentricities of the target, and also with a decrease in the saccadic duration for far targets. Levels of anxiety in stroke patients are manifested as lower inhibitory control, breaking the central fixation in the first 300ms of presenting an eccentric target flash. This is consistent with the findings of previous studies about eye movement in anxiety disorder [52,53,54]. Anxiety compromises the velocity profile of memory-guided saccades.

The higher the depression score in stroke patients, the greater the percentage of early and total error rates, suggesting that depression in stroke patients can impact the ability to inhibit reflexive saccade toward visual distractors. In line with previous research, depression limits the capacity to inhibit reflexive behaviors, mainly due to a dysfunction in the dorsolateral prefrontal cortex [55,56,57].

Our study revealed significant correlations between the time to complete the Color Trails Tests (CTT-1 and CTT-2) and the parameters of memory-guided saccades. Specifically, we observed that longer completion times were associated with higher mean and peak velocities in memory-guided saccades towards a near target. On the other hand, the time to complete the CTT-2 test correlated negatively with the mean velocity for a more eccentric target. This suggests that the longer it takes to finish the CTT-2 test, the slower the mean velocity of the saccades towards more eccentric targets, indicating a greater difficulty in processing and executing these movements. Impaired motor control and coordination, processing speed, and visuo-spatial abilities demonstrated by the higher time to complete the color trails evaluations suggest a differentiated impact on the velocity of the memory-guided saccade, underlining a possible dependance on remembered target location. This confirms previous research showing that memory-guided saccades mirror processing speed capabilities [57,58,59].

Parameters of memory-guided saccades towards more eccentric targets correlated with the number of errors made in both CTT-1 and CTT-2 tests. A higher number of errors at CTT-1 was correlated with shorter latencies, mean velocities, and peak velocities of the saccades. Additionally, the higher the number of errors during CTT-2, the lower the peak velocity of the saccades. For memory-guided saccades towards near targets, latency showed a positive correlation, with near misses on the CTT-1 test. Our findings underline that the velocity profiles of memory-guided saccades correlate with the number of errors made during CTTs, concurring with other research suggesting that attention and executive functions affect the planning of efficient memory-guided saccades [60,61,62].

Higher error rates were correlated with lower values of visually guided saccade (VGS) parameters for near targets, including mean velocity, peak velocity, and amplitude. A higher number of near misses was correlated with a higher percentage of visually guided saccades. For more eccentric targets, higher error rates were correlated with lower amplitudes. More near misses were correlated with higher amplitude, longer duration, higher mean velocity, and longer times to reach peak velocity for visually guided saccades. Error rates and near misses during the CTT were correlated with the processing of visually guided saccades performed to correct landing on the remembered target location with the actual target, indicating impaired inhibitory control. Supporting these findings, the TMT, a basic version of the Color Trails Test, has been shown to involve inhibitory control in addition to working memory [63,64]. Finally, the number of prompts during both CTT-1 and CTT-2 tests was found to correlate with a higher percentage of early errors. Interestingly, prompts during the CTT-1 test also correlated with a lower percentage of correctly executed memory-guided saccades. Individuals who require additional prompts during the Color Trails Test may be more susceptible to making early errors in the MGS task due to difficulties with inhibitory control.

An overall improved performance on the digit span test is associated with more accurate memory-guided saccades and faster corrective visually guided saccades. This indicates enhanced working memory and heightened attention in the evaluated subjects, supporting current research on eye tracking memory-guided tasks [65].

Individuals with higher symbol search scores show superior cognitive and perceptual abilities, including faster information processing, improved visual perception, and enhanced visuo-motor coordination [66]. This is reflected in more controlled and efficient eye movements during the memory-guided task. Higher incorrect identification rates indicate a less efficient corrective visually guided saccade and potentially less effective attention and working memory, underscoring the link between cognitive processes, visual perception, and eye movement behaviors in complex saccadic tasks [67].

Several limitations impacted our study: a small sample size (n = 62) of a heterogeneous disease, a lack of stroke severity categorization, a wide inclusion interval of 30 to 120 days post stroke, and data from a single evaluation. Additionally, we did not account for lesion sites or pre-stroke cognitive state. However, our work lays a foundation for future research that can address these limitations through larger studies that can validate and extend our findings.

## 5. Conclusions

While our research identified some correlations between cognitive measures and eye-tracking metrics during the Memory-Guided Saccadic Task, these relationships were not robust, potentially due to population heterogeneity. Nevertheless, these findings provide important insights into the complex interactions between cognitive status, psychological well-being, and oculomotor behavior. Future research should consider longitudinal designs to better understand the causal relationships and potential interventions to mitigate the effects of aging on memory-guided saccade performance. Additional studies may help in establishing Memory-Guided Saccadic Tasks as a clinically relevant tool for the cognitive functional assessment of stroke patients.

## Figures and Tables

**Table 2:** Additional memory-guided saccadic performance indicators [22,28].

Metric	Description
%MGS	% of correctly executed memory-guided saccades towards the remembered target location from the total number of trials
%CorrVGS	% of corrective visually guided saccades made toward the confirmation target location from the total number of valid trials
%EarlyErrorRate	% early errors—saccades made during the first 300 ms of the memorisation phase from the total number of valid trials
%LateErrorRate	% of late errors—saccades made during the second part of the memorisation phase (>301 ms) from the total number of valid trials
%TotalErrorRate	Total % saccades made during the memorisation phase from the total number of valid trials

**Table 3:** Descriptive statistics for participant age at stroke onset.

Age at Stroke Onset	N. of Patients/%	Mean	Median	SD
All subjects	62 (100%)	59.94	61.5	10.87664
Female	14 (22.59%)	61.92	63	9.12700
Male	48 (77.41%)	59.23	60.5	11.34983

**Table 4:** Correlation analysis results.

Variables of Interest	Eye-Tracking Parameter	Correlation Coefficient (ρ)	*p* Value
Age	%LateErrorRate	0.327	0.009
	%MGS	−0.274	0.003
	Time to Peak Velocity of NEAR VGS	0.273	0.035
MoCA (Montreal Cognitive Assessment)	Mean Duration of NEAR VGS	0.259	0.045
HADS-A (Anxiety score)	%EarlyErrorRate	0.325	0.01
	Mean Velocity of NEAR MGS	0.226	0.037
	Peak Velocity of NEAR MGS	0.279	0.028
	Peak Velocity of MGS FAR	0.259	0.042
	Duration of MGS FAR (SD)	−0.262	0.04
HADS-D (Depression score)	%EarlyErrorRate	0.331	0.014
	%TotalErrorRate	0.294	0.021
CLRES01-1 (Color Trails 1—time in seconds)	Mean Velocity of MGS NEAR (SD)	0.31	0.014
	Peak Velocity of MGS NEAR (SD)	0.21	0.09
CLRES02-1 (Color Trails 1—number of errors)	Latency of MGS FAR (SD)	−0.373	0.003
	Mean Velocity of MGS FAR (SD)	−0.324	0.01
	Peak Velocity of MGS FAR (SD)	−0.282	0.026
	Mean Velocity of VGS NEAR	−0.268	0.038
	Peak Velocity of VGS NEAR (SD)	−0.293	0.023
	Amplitude of VGS NEAR (SD)	−0.408	0.001
	Amplitude of VGS FAR (SD)	−0.302	0.019
CLRES03-1 (Color Trails 1—near misses)	Latency of MGS NEAR	0.236	0.065
	Peak Velocity of MGS FAR	−0.288	0.023
	%CorrVGS	0.315	0.013
	Amplitude of VGS FAR	0.313	0.014
	Mean Velocity of VGS FAR	0.335	0.008
	Time to Peak Velocity of VGS FAR	0.391	0.002
	Duration of VGS FAR	0.328	0.001
CLRES04-1 (Color Trails 1—number of prompts)	%EarlyErrorRate	0.275	0.03
	%MGS	−0.256	0.045
CLRES05-1 (Color Trails 2—time in seconds)	Mean Velocity of MGS NEAR(SD)	0.324	0.01
	Mean Velocity of MGS FAR	−0.343	0.006
CLRES06-1 (Color Trails 2—number of color errors)	Peak Velocity of MGS FAR	−0.41	<0.001
	Peak Velocity of MGS FAR (SD)	−0.373	0.003
CLRES09-1 (Color Trails 2—number of prompts)	%EarlyErrorRate	0.301	0.018
DGFRES-1 (Digit Span Forward)	Amplitude of MGS NEAR (SD)	0.258	0.043
	Gain of MGS NEAR (SD)	0.673	0.055
DGBRES-1 (Digit Span Backward)	Time to Peak Velocity of MGS NEAR	−0.387	0.002
	%CorrVGS	0.353	0.005
PSSCNUM-1 (Processing Speed—number correct)	Time to Peak Velocity of MGS NEAR	−0.26	0.042
	Mean Velocity of MGS NEAR (SD)	−0.284	0.025
	Latency of MGS FAR (SD)	−0.264	0.038
PSSINUM-1 (Processing Speed—number incorrect)	Duration of MGS NEAR (SD)	−0.229	0.074
	Time to Peak Velocity of VGS NEAR	0.34	0.008

**Table 5:** Multiple regression analysis results.

Predictor	B	SE	t	*p*
Intercept	−35.603	40.089	−0.888	0.378
Age	0.634	0.259	2.447	0.018
Gender	10.561	6.053	1.745	0.087
MOTS-1	0.103	1.015	0.101	0.920
HDTSA-1	0.349	1.096	0.319	0.751
HDTSD-1	−0.555	0.964	−0.576	0.567
CLRES01-1	0.066	0.155	0.422	0.675
CLRES02-1	−8.840	5.703	−1.550	0.127

## Data Availability

Replication Data: The N-PEP-12 clinical study’s secondary data analysis for Memory-Guided Saccadic Task eye-tracking evaluation in subacute and chronic stroke is available at https://doi.org/10.7910/DVN/VQ2AEE, accessed on 20 June 2024.

## Supplementary Materials

The following supporting information can be downloaded at: https://www.mdpi.com/article/10.3390/biomedicines12081678/s1, Table S1: Neuropsychological assessment scores; Table S2: Memory guided saccades performance indicators; Table S3: Parameters of memory guided saccades—descriptive statistics; Table S4: Parameters of visually guided saccades—descriptive statistics.
