# Supplementary material for: Memory-Guided Saccades in Subacute and Chronic Stroke: Secondary Data Analysis of the N-PEP-12 Clinical Study
Source: Biomedicines. 2024 Jul 26;12(8):1678. doi: 10.3390/biomedicines12081678 (PMC11351517; doi:10.3390/biomedicines12081678)
Supplement: Supplementary file 1 [file biomedicines-12-01678-s001.zip › biomedicines-3092800-supplementary.pdf]

Supplementary Materials

Memory Guided Saccades in Subacute and Chronic Stroke: Secondary Data Analysis of the N-PEP-12 Clinical Study

Table S1. Neuropsychological assessment scores.

Variable	Mean	Std.Dev.	Minimum	Maximum	Median	Rage
Moca – Total Score	23.62	3.47	13	29	24	16
HADS – Anxiety	5.35	3.41	0	14	5	14
HADS – Depression	4.56	3.86	0	15	4	15
CLRES01-1 (Colors Trails 1 - Time in Seconds)	67.53	20.53	30	144	62	114
CLRES02-1 (Colors Trails 1 – Errors)	0.14	0.47	0	3	0	3
CLRES03-1 (Colors Trails 1 - Near Miss)	0.16	0.41	0	2	0	2
CLRES04-1 (Colors Trails 1 – Prompts)	0.06	0.39	0	3	0	3
CLRES05-1 (Colors Trails 2 - Time in Seconds)	121.37	42.91	52	242	107.5	190
CLRES06-1 (Colors Trails 2 - Color Errors)	0.67	1.08	0	4	0	4
CLRES07-1 (Colors Trails 2 -Number Errors)	0.08	0.27	0	1	0	1
CLRES08-1 (Colors Trails 2 - Near Miss)	0.20	0.44	0	2	0	2
CLRES09-1 (Colors Trails 2 – Prompts)	0.08	0.41	0	3	0	3
Digit Span Forward (Digit Span - Digit Forward Total Score)	9.79	9.60	2	81	8	79
Digit Span Backward (Digit Span - Digit Backward Total Score)	5.80	2.86	1	13	5	12
PSI-Digit Symbol (PSI - Digit Symbol Coding - Number Correct)	52.95	19.14	20	90	53.5	70
PSI-Symbol Search - Number Correct	24.75	11.15	7	75	24.5	68
PSI-Symbol Search - Number Incorrect	1	1.37	0	5	0	5

Table S2. Memory guided saccades performance indicators.

Variable	Mean	Std. Dev.	Minimum	Maximum	Median	Range
%EarlyErrorRate	65.93	69.33	0	327.27	36.47	327.27
%LateErrorRate	20.18	20.17	0	77.77	12.90	77.77
%TotalErrorRate	86.12	76.07	2.94	333.33	61.80	33.39
%MGS	64.63	23.41	17.5	97.5	67.5	80
%CorrVGS	69.46	27.93	0	100	78.54	100

Table S3. Parameters of memory guided saccades - descriptive statistics.

Stimulus location	Variable	MEAN	MEAN	MEAN	MEAN	MEAN	MEAN	SD	SD	SD	SD	SD	SD
		Mean	SD	MIMIMUM	MAXIMUM	MEDIAN	RANGE	Mean	SD	MIMIMUM	MAXIMUM	MEDIAN	RANGE
NEAR	Latency	577.44	179.22	253.63	1243.63	550.44	990	290.74	133.79	70.79	683.11	292.81	612.32
	Amplitude	6.44	2.02	2.06	9.89	6.44	7.83	2.65	1.62	0.33	9.86	2.33	9.52
	Duration	66.42	16.44	30	110.5	69.83	80.5	17.50	13.11	2.30	88.77	14.89	86.46
	Mean Velocity	97.16	26.24	54.16	248.61	91.40	194.44	35.54	65.33	4.05	527.32	23.91	523.27
	Peak Velocity	363.84	218.38	142.33	1916.44	326.24	1674.11	180.24	521.82	28.83	4109.27	73.49	4080.44
	Time to Peak Velocity	32.22	6.76	17	60.27	31.28	43.27	9.07	7.66	0.97	34.18	6.39	33.21

	Gain	64.42	20.25	20.67	98.99	64.46	78.32	26.54	16.27	3.37	98.67	23.33	95.29
FAR	Latency	588.00	206.60	295.33	1217.5	534.57	922.16	284.95	222.72	16.26	1570.48	249.75	1554.22
	Amplitude	10.99	4.91	1.89	17.64	11.62	15.74	4.16	1.89	0.55	8.61	4.39	8.06
	Duration	87.14	29.13	31	158.10	91.17	127.10	27.12	17.96	1.41	93.33	22.74	91.92
	Mean Velocity	120.92	32.62	49.48	180.98	121.53	131.50	34.17	16.48	3.52	94.00	36.52	90.47
	Peak Velocity	396.09	154.87	143.76	1241.12	367.85	1097.35	186.56	265.51	25.21	1366.45	99.39	1341.24
	Time to Peak Velocity	39.49	13.44	12	84	35.01	72	15.66	14.98	0	73.53	9.64	73.53
	Gain	61.11	27.26	10.54	98.02	64.58	87.47	23.01	10.57	2.89	47.88	24.41	44.98

Table S4. Parameters of visually guided saccades – descriptive statistics.

**Stimulus location**	**Variable**	**MEAN**	**MEAN**	**MEAN**	**MEAN**	**MEAN**	**MEAN**	**SD**	**SD**	**SD**	**SD**	**SD**	**SD**
		Mean	SD	MIMIMUM	MAXIMUM	MEDIAN	RANGE	Mean	SD	MIMIMUM	MAXIMUM	MEDIAN	RANGE
NEAR	Latency	545.78	397.96	147.5	1896.5	410.27	1749	325.91	231.68	1.41	918.30	256.38	916.88
	Amplitude	6.21	2.61	1.68	12.74	5.62	11.05	2.66	1.33	0.07	6.36	2.69	6.29
	Duration	60.63	15.87	29.54	110	60.66	80.45	17.31	12.03	2.82	60.61	14.60	57.79
	Mean Velocity	99.94	33.25	44.32	225.39	99.72	181.07	32.42	17.54	1.71	81.57	28.01	79.85
	Peak Velocity	381.08	131.68	170.06	701.84	360.96	531.77	135.35	121.81	12.20	669.50	100.81	657.30
	Time to Peak Velocity	31.76	6.15	20.5	58.6	31.88	38.1	7.67	5.66	0.70	27.01	6.09	26.30
FAR	Latency	623.47	373.76	165.27	1611.4	496.8	1446.12	377.50	255.48	28.28	860.52	376.87	832.244
	Amplitude	9.66	5.17	2.20	25.48	8.78	23.28	4.62	2.37	0.27	10.66	4.63	10.39
	Duration	72.07	23.82	32	140	70.22	108	25.90	19.35	1.76	97.53	21.02	95.77
	Mean Velocity	124.47	39.58	54.08	205.40	125.71	151.32	43.92	25.44	4.92	117.98	40.40	113.05
	Peak Velocity	447.67	228.76	155.27	1441.91	386.35	1286.64	208.96	362.24	3.93	2074.43	111.35	2070.49
	Time to Peak Velocity	36.36	11.51	17.33	76.72	35.33	59.39	14.30	13.82	1.63	70.63	8.69	69.00
